# Association Between the Cholesterol–HDL–Glucose (CHG) Index and 90‐Day Functional Outcomes in Patients with Acute Ischemic Stroke

**DOI:** 10.1002/brb3.71505

**Published:** 2026-06-03

**Authors:** Xinyue Yuan, Xiangqi Kong, Mina Zhao, Penghong Li, Haobo Wang, Wei Jing

**Affiliations:** ^1^ Third Hospital of Shanxi Medical University, Shanxi Bethune Hospital, Shanxi Academy of Medical Sciences, Tongji Shanxi Hospital Taiyuan China

**Keywords:** acute ischemic stroke, CHG index, metabolic stress, modified Rankin Scale, prognosis

## Abstract

**Background**: Acute ischemic stroke (AIS) is a major cause of global disability and mortality. Metabolic disturbances, such as insulin resistance (IR) and abnormal lipid profiles, are linked to poor outcomes. The novel cholesterol, high‐density lipoprotein (HDL), and glucose (CHG) index integrates glucose and lipid metabolism, but its prognostic significance in AIS remains unclear.

**Methods**: This retrospective analysis included 541 AIS patients admitted to Shanxi Bethune Hospital between October 2023 and December 2024, excluding those receiving reperfusion therapy or with severe comorbidities. The CHG index was calculated on admission as CHG = ln{[(TC × FBG) / (2 × HDL)]× 18.02}. Unfavorable functional outcome at 90 days was defined as modified Rankin Scale >2.

**Results**: Among 541 patients, 193 (35.7%) had unfavorable outcomes. Elevated CHG levels were significantly associated with a progressively higher risk of unfavorable outcomes. Each one‐unit increase in CHG corresponded to 1.7516‐fold higher odds of unfavorable outcomes (OR = 1.7516). Quartile analysis showed that patients in the highest CHG quartile had 3.1277‐fold higher odds of unfavorable outcomes compared to the lowest quartile (*p* for trend <0.05). Restricted cubic spline analysis supported a linear association between CHG and unfavorable outcomes. Subgroup analyses revealed consistent associations across sex, smoking, alcohol use, hypertension, and diabetes.

**Conclusion**: The CHG index is an independent and easily accessible predictor of 90‐day functional outcomes in AIS. By capturing integrated metabolic stress, it may serve as a useful biomarker for early risk stratification.

## Graphical abstract

The CHG index, derived from total cholesterol, high‐density lipoprotein, and fasting blood glucose, was associated with 90‐day functional outcomes after acute ischemic stroke.

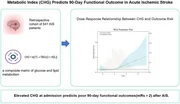

**Abbreviations**
AIS: acute ischemic stroke
BBB: blood–brain barrier
BMI: body mass index
CHG: cholesterol–high‐density lipoprotein–glucose index
CI: confidence interval
CT: computed tomography
FBG: fasting blood glucose
HDL: high‐density lipoprotein
IR: insulin resistance
METS‐IR: metabolic score for insulin resistance
MRI: magnetic resonance imaging
mRS: modified Rankin Scale
Neu: neutrophil
NIHSS: National Institutes of Health Stroke Scale
OR: odds ratio
RCS: restricted cubic spline
TC: total cholesterol
TG: triglycerides
TyG: triglyceride–glucose index
VIF: variance inflation factor

## 1 Introduction

As one of the primary causes of death and chronic disability globally, acute ischemic stroke (AIS) exerts a substantial burden on patients, caregivers, and healthcare systems (GBD 2021 Stroke Risk Factor Collaborators 2024). Despite advances in treatment and preventive strategies, the clinical course and functional recovery of stroke patients remain markedly heterogeneous (Ravipati et al. 2024; Kleindorfer et al. 2021). This variability is influenced by multiple interrelated factors. Among these factors, metabolic disturbances—especially insulin resistance (IR) and related lipid metabolism disorders—have been identified as key pathophysiological mechanisms that aggravate neurovascular injury and impair neural plasticity, ultimately influencing functional outcomes (Gao and Sun 2025). Traditionally, several surrogate markers have been developed to evaluate insulin resistance and metabolic stress, including the metabolic score for insulin resistance (METS‐IR) (Duan et al. 2024), the triglyceride–glucose (TyG) index (J. Chen, Wu, Lin, et al. 2023; Y. Chen et al. 2024), and the triglyceride‐to‐high‐density lipoprotein cholesterol ratio (TG/HDL) (Che et al. 2023). Although these markers show predictive value for cardiovascular and cerebrovascular diseases, their clinical applicability remains limited. For example, METS‐IR depends on body mass index (BMI) and does not directly measure lipid metabolic components. The TyG index does not incorporate HDL, an essential protective lipid component (Krishnamoorthy et al. 2025). Although the TG/HDL ratio effectively indicates atherogenic lipid patterns, it assesses only lipid metabolism. Therefore, a composite indicator that simultaneously integrates glucose, atherogenic lipids (total cholesterol, TC), and HDL may provide a more comprehensive evaluation of overall metabolic stress. Recently, a novel composite metabolic index—the cholesterol–HDL–glucose (CHG) index—has been proposed (Mansoori et al. 2025), defined as CHG = ln{[(TC × FBG) / (2 × HDL)] × 18.02}. Pathophysiologically, this index more accurately reflects the overall metabolic dysregulation characteristic of metabolic syndrome. Moreover, it overcomes the limitations of traditional markers by identifying stroke risk across a wider population spectrum, including individuals with normal TG/HDL ratio but elevated fasting glucose or abnormal total cholesterol levels.

Previous studies have shown that higher CHG serves as an independent predictor of metabolic syndrome (Wei et al. 2025), and type 2 diabetes (Çatak et al. 2025), and atherosclerotic cardiovascular disease (Mo et al. 2025). However, the relationship between CHG and stroke outcomes has not been systematically explored. It remains unclear whether elevated CHG levels are linked to adverse functional outcomes, recurrent stroke events, or mortality. Employing a retrospective cohort design, this study evaluated the link between admission CHG levels and 90‐day functional outcomes in AIS patients, defining poor outcomes as a modified Rankin Scale (mRS) score greater than 2. We hypothesized that elevated CHG levels represent an independent factor for 90‐day unfavorable events in AIS. These findings may confirm the prognostic utility of the CHG index in stroke and provide an effective early‐warning tool for risk stratification, thereby facilitating more individualized management strategies for patients with AIS.

## 2 Materials and Methods

### 2.1 Participants

This retrospective study included patients with AIS who were diagnosed in the Department of Neurology at Shanxi Bethune Hospital between October 2023 and December 2024. The inclusion of ischemic stroke cases was based on the diagnostic criteria established by the World Health Organization. All diagnoses were confirmed using brain magnetic resonance imaging (MRI) or computed tomography (CT).

**FIGURE 1:**
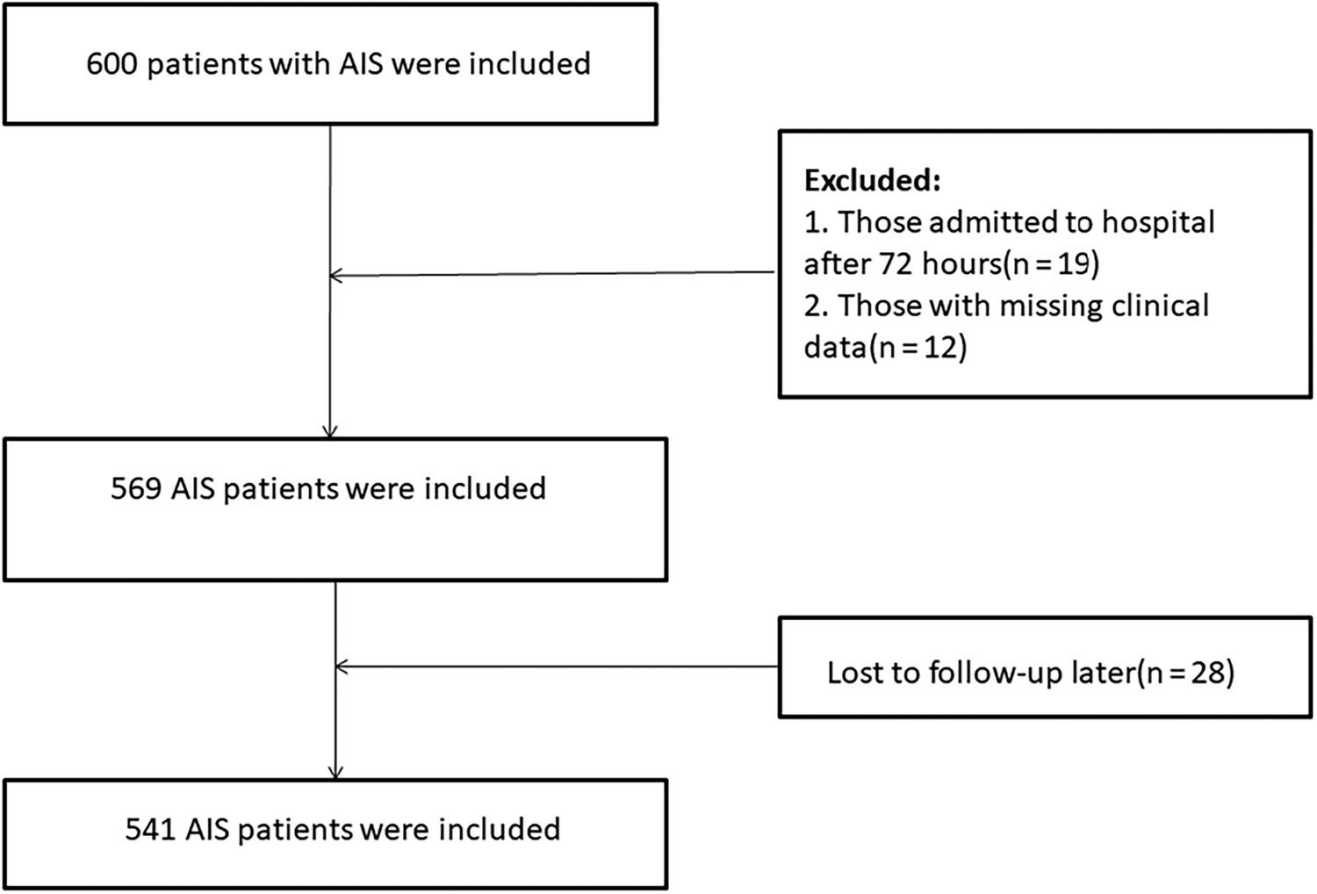
Flowchart of participant selection.

The inclusion criteria were as follows: (1) stroke confirmed by CT or MRI within 72 h of onset, consistent with the 2018 Chinese Guidelines for the Diagnosis and Treatment of AIS; (2) first‐ever stroke with no prior severe neurological deficits; (3) age ≥18 years; and (4) complete clinical data with informed consent provided. The exclusion criteria were as follows: (1) diagnosis of transient ischemic attack or hemorrhagic cerebrovascular disease; (2) history of psychiatric disorders (clinically diagnosed or previously treated); (3) severe aphasia or disturbances of consciousness preventing participation in assessments due to visual, auditory, or comprehension impairments; (4) severe cardiopulmonary disease or multi‐organ failure; (5) chronic inflammatory diseases; (6) incomplete clinical data; (7) loss to follow‐up; and (8) having undergone reperfusion therapy (including intravenous thrombolysis and/or endovascular intervention). A total of 541 eligible participants were included in the final analysis (Figure 1).

### 2.2 Data Collection

Venous blood samples (2 mL) were collected in EDTA‐K2 anticoagulant tubes, gently mixed, and analyzed using a fully automated hematology analyzer (Beckman DXH800) to measure white blood cell, neutrophil (Neu), monocyte, lymphocyte, and platelet counts. Additional venous blood samples (5 mL) were collected into serum separator tubes without anticoagulant and centrifuged to measure serum uric acid, TC, triglycerides (TG), HDL, low‐density lipoprotein, and homocysteine levels. D‐dimer levels were determined in citrated plasma (blood‐to‐citrate ratio 9:1) using a WoFen ACL‐TOP750 analyzer. In addition, fasting blood glucose samples were obtained from all patients. All fasting blood specimens were collected in accordance with standard clinical laboratory protocols after at least 8 h of fasting following hospital admission, typically overnight.

### 2.3 Prognostic Assessment

Functional outcome at 90 days, assessed by mRS, served as the primary endpoint. A favorable outcome was defined as an mRS score of ≤2, whereas an unfavorable outcome was defined as an mRS score of >2.

### 2.4 CHG Index Calculation

In this study, the CHG index, as defined by Mansoori et al. (2025), was used to assess individual metabolic risk. Because lipid and glucose measurements in our laboratory were reported in millimoles per liter, while the original formula requires milligrams per deciliter, the following conversion factors were applied: TC, 1 mmol/L = 38.67 mg/dL; fasting blood glucose (FBG), 1 mmol/L = 18.02 mg/dL; and HDL, 1 mmol/L = 38.67 mg/dL. After conversion, the original formula was applied: CHG = ln((TC × FBG)/(2 × HDL)). For convenience, the conversion factor was incorporated into the formula, yielding: CHG = ln{[(TC × FBG)/(2 × HDL)]× 18.02}. This adjusted formula was applied for all CHG calculations in the present study. Notably, the CHG index is a dimensionless composite metabolic indicator derived from logarithmic transformation.

### 2.5 Statistical Analysis

Baseline characteristics were compared between the groups according to 90‐day functional outcomes. The Shapiro–Wilk test was used to assess the normality of continuous variables. Variables with a normal distribution were reported as mean ± standard deviation, whereas non‐normally distributed variables were presented as median (interquartile range, 25th–75th percentiles). Between‐group differences were assessed using the Student's *t*‐test or the Mann–Whitney *U* test, as appropriate. Categorical variables were reported as frequencies and percentages, and group differences were analyzed using the chi‐square test.

Multivariable logistic regression analyses were conducted to examine the correlation between the CHG index and unfavorable functional outcomes. Potential confounders were identified according to clinical knowledge, previous literature, and univariable analysis results. Three models were constructed: Model 1 was unadjusted; Model 2 was adjusted for demographic variables, including sex, smoking status, and alcohol consumption; and Model 3 was further adjusted for clinically relevant variables, including National Institutes of Health Stroke Scale (NIHSS) score, BMI, Neu, and D‐dimer levels.

To mitigate potential multicollinearity and enhance the stability of the regression models, Spearman rank correlation analysis and variance inflation factor (VIF) testing were conducted. Spearman analysis demonstrated a strong negative correlation between CHG and HDL (*ρ* = –0.955, *p* < 0.001); consequently, HDL was excluded from the final model. Subsequent VIF testing (see Table S2) indicated that all covariates included in the final model had VIF values below 2, suggesting no significant multicollinearity.

Sensitivity analyses were conducted by categorizing the CHG index into quartiles to examine linear trends and assess the robustness of the findings. Restricted cubic spline (RCS) models with four knots were employed to explore potential nonlinear associations between the CHG index and unfavorable outcomes. Subgroup analyses and interaction tests were subsequently performed across strata of sex, smoking status, alcohol consumption, hypertension, and diabetes. Each subgroup analysis was adjusted for the same covariates included in Model 3, excluding the variable defining the subgroup. All statistical analyses were conducted using R Studio (version 4.4.2) and DecisionLinc 1.0, and a two‐tailed *p*‐value < 0.05 was regarded as indicative of statistical significance.

## 3 Results

### 3.1 Baseline Characteristics of Participants

The study included a total of 541 patients, comprising 348 with favorable outcomes and 193 with unfavorable outcomes (Table 1). Significant between‐group differences were observed in sex, smoking and drinking status, NIHSS score, D‐dimer levels, Neu, HDL, and the CHG index (all *p* < 0.05). Specifically, patients with unfavorable outcomes were more often male, smoked and drank more frequently, and exhibited higher NIHSS scores and D‐dimer levels, whereas HDL levels were significantly lower. Furthermore, the CHG index was elevated in the unfavorable outcome group.

**TABLE 1:** Baseline characteristics of participants.

Characteristic	Overall (N = 541)	Favorable outcome (N = 348)	Unfavorable outcome (N = 193)	*p*‐value^a^
Sex, n (%)				<0.001
Female	162 (30)	131 (38)	31 (16)	
Male	379 (70)	217 (62)	162 (84)	
Education, n (%)				0.708
Illiteracy	88 (16)	59 (17)	29 (15)	
Primary school	193 (36)	118 (34)	75 (39)	
Junior high school	218 (40)	143 (41)	75 (39)	
Senior high school and above	42 (7.8)	28 (8.0)	14 (7.3)	
Smoking, n (%)				<0.001
No	226 (42)	165 (47)	61 (32)	
Yes	315 (58)	183 (53)	132 (68)	
Drinking, n (%)				<0.001
No	271 (50)	193 (55)	78 (40)	
Yes	270 (50)	155 (45)	115 (60)	
HTN, n (%)				0.636
No	217 (40)	137 (39)	80 (41)	
Yes	324 (60)	211 (61)	113 (59)	
DM, n (%)				0.541
No	376 (70)	245 (70)	131 (68)	
Yes	165 (30)	103 (30)	62 (32)	
CAD, n (%)				0.438
No	495 (91)	316 (91)	179 (93)	
Yes	46 (8.5)	32 (9.2)	14 (7.3)	
Lesion location, n (%)				0.062
Basal ganglia or lateral ventricles	237 (44)	142 (41)	95 (49)	
Brain stem or cerebellum	152 (28)	106 (30)	46 (24)	
Thalamus	15 (2.8)	12 (3.4)	3 (1.6)	
Cerebral lobe	35 (6.5)	27 (7.8)	8 (4.1)	
Multiple infarction	102 (19)	61 (18)	41 (21)	
Stroke location, n (%)				0.358
Left	339 (63)	213 (61)	126 (65)	
Right	180 (33)	118 (34)	62 (32)	
Both	22 (4.1)	17 (4.9)	5 (2.6)	
NIHSS, median (IQR)	3.00 (1.00, 4.00)	2.00 (1.00, 3.00)	4.00 (3.00, 5.00)	<0.001
Age (years), median (IQR)	63.00 (55.00, 70.00)	62.00 (55.00, 70.00)	63.00 (56.00, 70.00)	0.916
BMI (kg/m^a^), median (IQR)	22.44 (18.63, 27.09)	22.16 (18.34, 26.72)	23.15 (19.64, 27.74)	0.055
WBC (×10^9^/L), median (IQR)	6.40 (5.30, 7.70)	6.40 (5.20, 7.70)	6.40 (5.53, 7.90)	0.329
Lym (×10^9^/L), median (IQR)	2.53 (1.97, 3.36)	2.54 (1.98, 3.38)	2.52 (1.91, 3.32)	0.473
Mon (×10^9^/L), median (IQR)	0.53 (0.44, 0.65)	0.52 (0.43, 0.65)	0.55 (0.46, 0.65)	0.302
Neu (×10^9^/L), median (IQR)	5.94 (4.77, 6.89)	6.02 (5.16, 6.89)	5.70 (3.42, 6.86)	0.029
PLT (×10^9^/L), median (IQR)	238.76 (194.28, 284.48)	235.97 (194.59, 284.20)	241.47 (192.64, 285.27)	0.462
D‐dimer (ng/mL), median (IQR)	125.00 (77.00, 253.00)	112.00 (70.00, 193.50)	178.00 (94.00, 354.00)	<0.001
UA (mmol/L), median (IQR)	299.00 (244.40, 386.20)	294.90 (240.15, 378.30)	305.60 (255.30, 393.60)	0.073
FPG (mmol/L), median (IQR)	5.60 (4.94, 6.76)	5.59 (4.91, 6.65)	5.68 (5.06, 7.03)	0.373
TG (mmol/L), median (IQR)	1.48 (1.22, 1.75)	1.47 (1.17, 1.82)	1.49 (1.26, 1.72)	0.984
TC (mmol/L), median (IQR)	4.16 (3.47, 4.88)	4.16 (3.51, 4.98)	4.15 (3.44, 4.77)	0.365
LDL (mmol/L), median (IQR)	2.66 (2.22, 3.18)	2.61 (2.19, 3.27)	2.68 (2.27, 3.10)	0.440
Hcy (µmol/L), median (IQR)	16.60 (12.90, 26.50)	16.20 (12.90, 25.80)	17.60 (12.90, 27.10)	0.413
HDL (mmol/L), median (IQR)	0.93 (0.80, 1.10)	0.99 (0.87, 1.16)	0.83 (0.74, 0.96)	<0.001
CHG, median (IQR)	3.28 (2.73, 3.76)	3.05 (2.58, 3.55)	3.65 (3.20, 4.21)	<0.001

Abbreviations: CAD: coronary artery disease; CHG: cholesterol, high‐density lipoprotein, and glucose index; DM: diabetes mellitus; FPG: fasting plasma glucose; Hcy: homocysteine; HDL: high‐density lipoprotein; HTN: hypertension; LDL: low‐density lipoprotein; Lym: lymphocyte; Mon: monocyte; Neu: neutrophil; PLT: platelet counts; TC: total cholesterol; TG: triglycerides; UA: uric acid; WBC: white blood cell.

^a^ Pearson's chi‐squared test; Wilcoxon rank sum test.

### 3.2 Association Between CHG Index and 90‐Day Functional Outcomes in AIS Patients

Univariable regression analysis (Table S1) indicated that sex, smoking status, alcohol consumption, NIHSS score, D‐dimer levels, BMI, Neu, HDL, and the CHG index were all significantly associated with 90‐day functional outcomes in patients with AIS. Multivariable logistic regression analysis demonstrated that higher CHG index levels were significantly linked to a higher risk of unfavorable 90‐day outcomes (Table 2). In the unadjusted model (Model 1), each 1‐unit increase in the CHG index was associated with an odds ratio (OR) of 2.4018 (95% confidence interval [CI]: 1.8964–3.0980, *p* < 0.0001). After adjusting for sex, smoking status, and alcohol consumption (Model 2), the association remained statistically significant (OR = 2.2977, 95% CI: 1.8031–2.9813). Further adjustment for NIHSS score, BMI, Neu, and D‐dimer levels (Model 3) resulted in a modest attenuation of the effect size; however, the association persisted and remained highly significant (OR = 1.7516, 95% CI: 1.3653–2.2970, *p* < 0.0001).

**TABLE 2:** Multivariable logistic regression analysis of the association between CHG and 90‐day functional prognosis of AIS.

Exposures	Model 1, OR (95% CI), *p*‐value	Model 2, OR (95% CI), *p*‐value	Model 3, OR (95% CI), *p*‐value
CHG	2.4018 (1.8964, 3.0980),<0.0001	2.2977 (1.8031, 2.9813), <0.0001	1.7516 (1.3653, 2.2970), <0.0001
Quartiles			
Q1	Reference	Reference	Reference
Q2	1.0564 (0.5835, 1.9155), 0.8558	0.9691 (0.5288, 1.7771), 0.9189	0.8681 (0.4259, 1.7672), 0.6958
Q3	3.0409 (1.7836, 5.2869), 0.0001	2.7437 (1.5801, 4.8505), 0.0004	2.3858 (1.2359, 4.6896), 0.0104
Q4	5.8721 (3.4478, 10.2488), <0.0001	5.2513 (3.0363, 9.2942), <0.0001	3.1277 (1.6275, 6.2193), 0.0007
*p* for trend	<0.0001	<0.0001	<0.0001

*Note*: Model 1  =  no covariates were adjusted. Model 2  =  Model 1 + sex, smoking, and drinking were adjusted. Model 3  =  Model 2 + NIHSS, BMI, Neu, and D‐dimer were adjusted.

When the CHG index was categorized into quartiles, trend analyses yielded consistent results. Compared with the lowest quartile (Q1), patients in the highest quartile (Q4) had a 3.1277‐fold higher risk of unfavorable outcomes in Model 3 (OR = 3.1277, *p* = 0.0007). The trend test was highly significant (*p* < 0.001), indicating a clear dose–response relationship between the CHG index and unfavorable outcomes.

### 3.3 Assessment of Nonlinear Relationship

**FIGURE 2:**
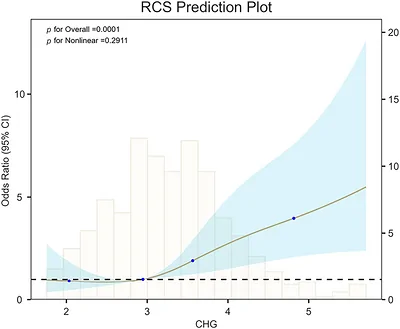
RCS showing the association between CHG and 90‐day functional outcomes.

RCS analysis (Figure 2) demonstrated that the risk of unfavorable functional outcomes in individuals with AIS increased steadily with rising CHG index levels, exhibiting a significant positive association (overall trend, *p* = 0.0001). Further testing for nonlinearity yielded a *p*‐value of 0.2911, which was not statistically significant, indicating that the association between the CHG index and 90‐day unfavorable outcomes followed a linear trend.

### 3.4 Subgroup Analyses

**FIGURE 3:**
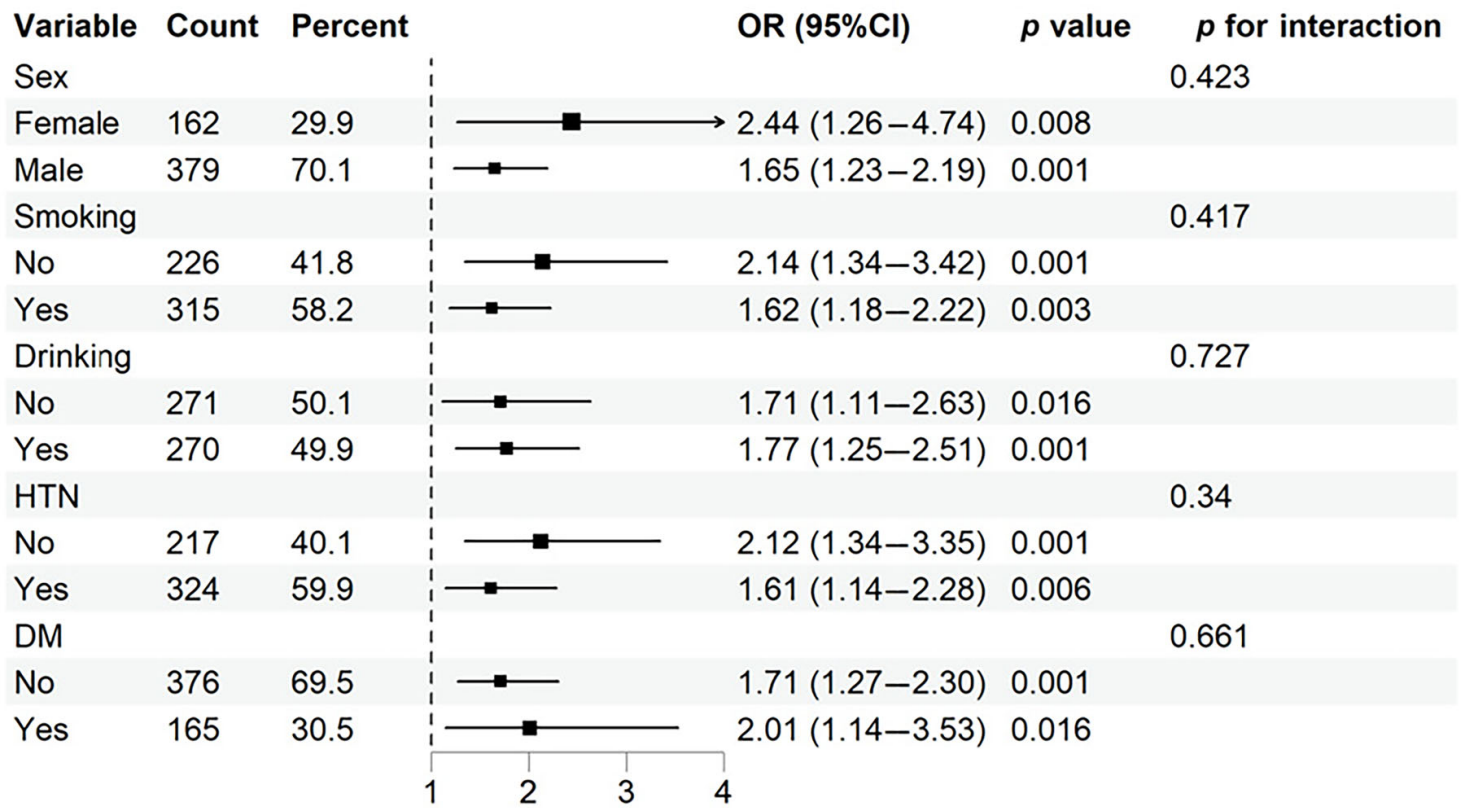
Subgroup analysis for the association between CHG and 90‐day functional outcomes.

Subgroup analyses were carried out by stratifying participants based on sex, smoking and alcohol use, and the presence or absence of hypertension and diabetes. As illustrated in Figure 3, no significant interactions were detected between the CHG index and any of the subgroups (interaction *p* > 0.05).

## 4 Discussion

This investigation included 541 AIS patients who did not receive reperfusion therapy. We present the first evidence that the admission CHG index is associated with 90‐day unfavorable functional outcomes (mRS > 2) in a clear dose–response relationship. After adjusting for sex, smoking status, alcohol consumption, stroke severity, comorbidities, and inflammatory markers, restricted cubic spline analysis showed no significant evidence of nonlinearity. Quartile trend analyses further indicated a progressive increase in risk with higher CHG index levels. Compared with Q1, individuals in Q4 had a 3.1277‐fold higher odds of unfavorable outcomes in Model 3 (OR = 3.1277). Subgroup analyses showed no significant interactions, indicating that the link between the CHG index and unfavorable outcomes remained consistent across all subgroups. The results address an important evidence gap regarding the prognostic significance of CHG for long‐term functional events in AIS and provide novel support for its potential role as an early risk stratification tool. This study is the first to explore the prognostic value of CHG in individuals with AIS.

In the context of this study, the CHG index represents an integrated metabolic burden resulting from the coexistence of glucose and lipid dysregulation rather than a single metabolic abnormality. Consistent with previous reports, elevated CHG levels are closely associated with metabolic syndrome, atherosclerotic cardiovascular disease, and type 2 diabetes, all of which are known to worsen stroke prognosis. Elevated fasting blood glucose reflects impaired metabolic regulation and is frequently accompanied by insulin resistance and increased oxidative stress. Experimental and clinical studies suggest that post‐stroke hyperglycemia disrupts mitochondrial energy metabolism in cerebral microvascular endothelial cells, thereby increasing blood–brain barrier(BBB) permeability and predisposing patients to hemorrhagic transformation (Xu et al. 2024). At the molecular level, hyperglycemia promotes the accumulation of reactive oxygen species and the activation of matrix metalloproteinases, which contribute to tight junction protein degradation and endothelial injury (Kim et al. 2024; Lochhead et al. 2024). In parallel, glucose‐derived metabolites, such as methylglyoxal, can activate the NLRP3 inflammasome and promote the release of proinflammatory cytokines, thereby further amplifying vascular inflammation and BBB dysfunction (Yang et al. 2024; Zhu et al. 2021; Wu et al. 2024).

Lipid dysregulation represents another critical component of this pathological network and interacts closely with glucose abnormalities. Elevated TC contributes to endothelial injury through the accumulation of oxidized lipoproteins, whereas reduced or dysfunctional HDL weakens protection against oxidative stress and inflammation. Papagiannis et al. (2023) demonstrated that impaired HDL cholesterol efflux capacity and reduced paraoxonase‐1 activity independently predict stroke severity and functional dependence, while excessively low cholesterol levels after reperfusion have also been associated with cerebral edema and BBB instability (Escudero‐Martínez et al. 2023). Importantly, disturbances in glucose and lipid metabolism reinforce one another, resulting in sustained vascular dysfunction and chronic inflammation that progressively damage the neurovascular unit. This adverse metabolic milieu suppresses brain‐derived neurotrophic factor expression, disrupts mitochondrial renewal, and impairs synaptic remodeling and myelin regeneration, thereby limiting post‐ischemic neural repair (Tuwar et al. 2023; M.‐B. Tang, Liu, et al. 2025; Rakshe et al. 2024). Experimental evidence further indicates that simultaneous modulation of glucose and lipid metabolism is more effective than targeting a single pathway (Zhao et al. 2021), supporting the notion that composite indices provide superior prognostic value compared with individual biomarkers (Jiang et al. 2024). By integrating TC, HDL, and FBG, the CHG index is able to capture this synergistic metabolic–vascular–neural cascade more comprehensively than individual biomarkers, offering a mechanistic explanation for its association with poor functional outcomes at 90 days after acute ischemic stroke (J. Tang, Wei, et al. 2025; Pacinella et al. 2024; Guo et al. 2022; G. Chen, Wu, Chen, et al. 2023).

The clinical implications of this study are noteworthy. The CHG index can be calculated directly from routine laboratory data without additional testing costs, making it highly suitable for early risk stratification. By identifying patients with elevated CHG at admission, clinicians can promptly recognize individuals at high risk of stroke recurrence and poor functional recovery, enabling early implementation of intensified metabolic management strategies, such as strict glucose and lipid control or lifestyle interventions. Moreover, the CHG index may serve as a valuable endpoint in clinical studies of metabolic interventions for stroke, providing an effective link between risk assessment and clinical outcome management.

Nevertheless, several limitations should be acknowledged. First, this was a single‐center retrospective study. Although multivariable analyses were employed to control for confounding factors, unmeasured confounders may remain, and caution is required when generalizing the findings. Second, we were unable to fully account for the effects of medications, including insulin, statins, and corticosteroids, which may introduce residual confounding related to lipid and glucose metabolism. Third, only single measurements of cholesterol, HDL, and glucose at admission were available, which precluded the assessment of their temporal dynamics and their relationship with outcomes. In addition, patients with severe stroke and those receiving reperfusion therapy were excluded, which may introduce potential selection bias and limit the generalizability of the findings to a broader acute ischemic stroke population. Finally, the underlying biological mechanisms linking the CHG index with prognosis remain unclear and require further investigation in basic research.

## 5 Conclusion

In summary, this study identifies the CHG index as a novel and readily obtainable biomarker that is associated with metabolic and inflammatory stress and independently predicts outcomes in patients with AIS, providing a more comprehensive evaluation of metabolic status in relation to cerebrovascular outcomes. Future studies should aim to confirm the predictive value of CHG across broader and more diverse cohorts and to explore its possible role in supporting individualized management of AIS.

## Author Contributions

**Xinyue Yuan**: writing – original draft, writing – review and editing. **Mina Zhao**: investigation. **Penghong Li**: investigation. **Haobo Wang**: investigation. **Wei Jing**: supervision, data curation, writing – review and editing. **Xiangqi Kong**: writing – original draft, software, methodology.

## Funding

This work was supported by Basic Research Programs of Science and Technology Commission Foundation of Shanxi Province (grant number 202503021211244), Cultivation Project for National Natural Science Foundation at Shanxi Bethune Hospital (2024GZRZ01) and Shanxi Medical University Education and Teaching Research Project（XJ2024025）.

## Ethics Statement

The studies involving humans were approved by Scientific Research Department of Shanxi Bethune Hospital (IRB Approval Number: YXLL‐2025‐231). The studies were conducted in accordance with local legislation and institutional requirements. Written informed consent for participation was not required from the participants in accordance with national legislation and institutional requirements.

## Conflicts of Interest

The authors declare no conflicts of interest.

## Supporting information




**Supplementary Table S1‐S2**: brb371505‐sup‐0001‐TableS1‐S2.docx

## Data Availability

The datasets used and analyzed during the current study are available from the corresponding author on reasonable request.
