# Supplementary material for: Association Between the Cholesterol–HDL–Glucose (CHG) Index and 90‐Day Functional Outcomes in Patients with Acute Ischemic Stroke
Source: Brain Behav. 2026 Jun 3;16(6):e71505. doi: 10.1002/brb3.71505 (PMC13580812; doi:10.1002/brb3.71505)
Supplement: Supplementary file 1 [file BRB3-16-e71505-s001.docx]

Catalogue

[Table S1 Univariate logistic regression analysis	2](#_Toc204433596)

[Table S2 Multicollinearity Diagnostics Using Variance Inflation Factor (VIF)	3](#_Toc204433597)

## Table S1 Univariate logistic regression analysis

| Variable Name | OR（95%CI） | P value |
| --- | --- | --- |
| NIHSS | 2.1688 （1.8829—2.5273） | 0.0000 |
| Sex(Male) | 3.1547 （2.0519—4.9710） | 0.0000 |
| Smoking | 2.0218 （1.4009—2.9407） | 0.0002 |
| drinking | 1.8978 （1.3299—2.7198） | 0.0004 |
| BMI | 1.0272 （1.0004—1.0549） | 0.0469 |
| Neu | 0.8742 （0.7964—0.9590） | 0.0045 |
| D-dimer | 1.0008 （1.0003—1.0014） | 0.0025 |
| CHG | 2.4018 （1.8964—3.0980） | 0.0000 |
| HDL | 0.0547 （0.0219—0.1279） | 0.0000 |
| Age | 0.9983 （0.9820—1.0150） | 0.8423 |
| Education  illiteracy  Primary schhool  junior high school  senior high school and above | — —  1.3620 (0.8037—2.3444)  1.1469 (0.6807—1.9628)  1.0714 (0.4820—2.3266) | —  0.2567  0.6110  0.8629 |
| DM | 1.0842 （0.7390—1.5832） | 0.6771 |
| CAD | 0.7723 （0.3901—1.4580） | 0.4390 |
| HTN | 0.8870 （0.6205—1.2701） | 0.5115 |
| WBC | 1.0533 （0.9852—1.1261） | 0.1261 |
| Lym | 0.8676 （0.7225—1.0306） | 0.1093 |
| Mon | 1.9829 （0.6818—5.7886） | 0.2086 |
| PLT | 1.0015 （0.9990—1.0040） | 2.2371 |
| UA | 1.0010 （0.9993—1.0028） | 0.2441 |
| TG | 0.9255 （0.6490—1.2981） | 0.6595 |
| FPG | 1.0109 （0.9968—1.0739） | 0.4747 |
| TC | 0.9077 （0.7647—1.0743） | 0.2634 |
| LDL | 1.0726 （0.8581—1.3406） | 0.5373 |
| Hcy | 1.0050 （0.9970—1.0133） | 0.2215 |
| LesionLocation  Basal ganglia or lateral ventricles  Brain stem or Cerebellum  Thalamus  cerebral lobe  Multiple infarction | — —  0.6487 (0.4187—0.9963)  0.3737 (0.0835—1.2137)  0.4429 (0.1814—0.9753)  1.0047 (0.6234—1.6097) | —  0.0500  0.1352  0.0546  0.9846 |
| StrokeLocation  Left  Right  Both | — —  0.8242 (0.5619—1.2021)  0.4828 (0.1564—1.2594) | —  0.3185  0.1646 |

## Table S2 Multicollinearity Diagnostics Using Variance Inflation Factor (VIF)

| Variable Name | VIF |
| --- | --- |
| NIHSS | 1.1689 |
| sex | 1.5226 |
| smoking | 1.5812 |
| Drinking | 1.4665 |
| BMI | 1.0127 |
| Neu | 1.0979 |
| D-dimer | 1.0311 |
| CHG | 1.0162 |
